# Polyploidy drives autophagy to participate in plant‐specific functions

**DOI:** 10.1002/imt2.252

**Published:** 2024-12-09

**Authors:** Moyang Liu, Ming Yang, Heng Liang, Bote Luo, Junjie Deng, Lingyan Cao, Daojun Zheng, Cheng Chen

**Affiliations:** ^1^ Shanghai Collaborative Innovation Center of Agri‐Seeds/School of Agriculture and Biology Shanghai Jiao Tong University Shanghai China; ^2^ Institute of Tropical Horticulture Research, Hainan Academy of Agricultural Sciences Haikou China; ^3^ Tropical Horticultural Plant Research Center, Hainan Research Institute Shanghai Jiao Tong University Sanya China; ^4^ Joint Center for Single Cell Biology, School of Agriculture and Biology Shanghai Jiao Tong University Shanghai China; ^5^ Joint International Research Laboratory of Metabolic and Developmental Sciences, State Key Laboratory of Hybrid Rice, School of Life Sciences and Biotechnology Shanghai Jiao Tong University Shanghai China

## Abstract

Polyploidization promotes the functional diversification of autophagy in plants, expanding autophagy‐associated genes (AAGs) to support processes like chloroplast division and flowering. Analysis of 92,967 AAGs in *Arabidopsis thaliana*, *Solanum lycopersicum, Camellia oleifera*, and 74 other plant species shows that 45.69% of AAGs are polyploidy‐related, highlighting polyploidy's role in linking autophagy to plant‐specific functions.

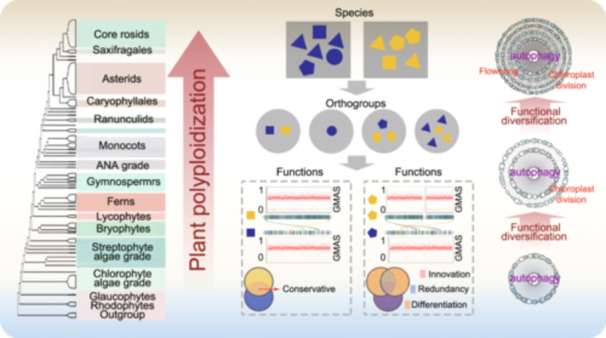

To the Editor,

Autophagy is a highly coordinated process, and its core mechanism is that autophagy proteins gradually form an autophagic vesicle, which encapsulates the substrate and transports it to a specific region for degradation [1]. Although the basic mechanisms are conserved in different eukaryotes, autophagy has gradually become involved in more biological processes during the course of evolution [1]. In single‐celled yeasts, autophagy is almost exclusively associated with the recycling of cellular materials [2]. The defects in autophagy impede processes such as organelle remodeling, synaptic transmission in neurons, and embryogenesis. In plants, autophagy is also associated with the processes of seedling development, leaves aging, and stress response, among others [3, 4]. Studies have shown that most plant autophagy genes can hardly rescue the functional defects caused by homologous knockouts in yeast, except for *autophagy‐regulated gene 4/6/8* (*ATG4/6/8*) [5, 6, 7]. How autophagy develops specific plant functions is still unclear. Here, we used a series of multiomics data and evolutionary strategies to investigate the evolutionary driver of plant‐specific autophagy‐associated functions.

We characterized the biological processes in which autophagy participates in plants by establishing a co‐functional network of autophagy. To this end, we obtained nearly 10,000 raw data samples from public databases such as Gene Expression Omnibus (GEO), ArrayExpress, and China National GeneBank DataBase (CNGBdb) (Figure S1A, Table S1), identifying autophagy‐associated genes (AAGs) at the levels of transcription, transcriptional regulation, and protein interactions. At the transcript level, we used the Gene‐Module Association Determination (G‐MAD) tool [8] to identify 10,964 genes which associated with the autophagy (Gene Ontology [GO]: 0006914, GO:0006914) (Figure 1A, Table S2). At the transcriptional regulation level, we identified 4757 genes associated with autophagy (GO: 0006914) based on DNA affinity purification sequencing (DAP‐seq) [12] (Figure 1B, Table S3). At the protein interactions level, we identified 12,675 genes associated with autophagy (GO: 0006914) based on Co‐fractionation MS (CF‐MS) databases [13] (Figure 1C, Table S4).

**Figure 1:**
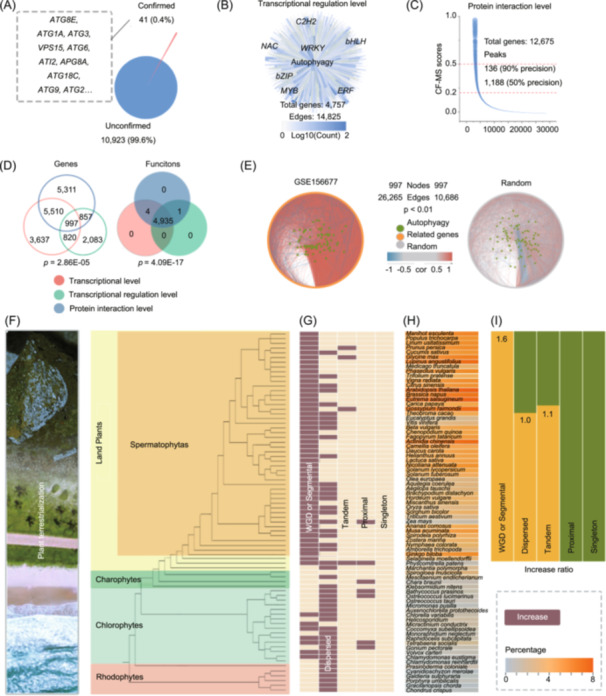
Co‐functional network of plant autophagy. (A) Autophagy‐associated genes (AAGs) were obtained using Gene‐Module Association Determination (G‐MAD). (B) Transcription factors involved in autophagy were identified using DNA affinity purification sequencing (DAP‐seq). (C) Genes involved in autophagy were identified using co‐fractionation MS (CF‐MS). (D) Venn diagram of genes and functions related to autophagy at the transcription, protein, and transcriptional regulation levels. (E) Correlation network of Core AAGs and autophagy data set (GSE156677) or random data set. Statistical significance was determined by comparing the complexity of the network (the number of edges) and the degree of correlation (the color of edges) (*p* < 0.01). (F) Plant phylogeny, based on previous studies [9, 10]. (G) The percentages of AAGs across all annotated genes in each genome. The more saturated the color, the higher the percentage. (H) Each gene classification is displayed with a heatmap [11], with significance levels relative to genome‐wide averages shown in different colors: enriched (red, *p* < 0.01), or not significantly different (yellow). (I) The ratio of each gene classification in terrestrial plants: increased (yellow, *p* < 0.01), or not significantly different (green).

Based on the results of G‐MAD, the Module‐Module Association Determination (M‐MAD) tool was employed to establish connections between autophagy and other biological functions, and the plant autophagy co‐functional network was constructed according to the obtained module‐module association scores (MMASs) (Table S5). Notably, the overlap analysis indicated that although genes associated with autophagy differed dramatically at different levels, they tended to perform similar functions. These genes included 1490 with biological functions known to be associated with autophagy, such as regulation of mRNA processing, negative regulation of growth, and positive regulation of programmed cell death, as well as 3419 with potential biological functions not previously associated with autophagy, such as regulation of cell growth by extracellular stimulus, positive regulation of phytol biosynthetic process and sequestering of iron ion (Figure 1D, Table S5). Further in the co‐function network, transcription, transcription regulation, and protein levels share 997 AAGs (Figure 1D), which are significantly correlated with the autophagy data set (GSE156677) and not significantly correlated with the random data set (Figure 1E, Table S6). Therefore, these 997 genes are defined as core AAGs.

To reveal the evolutionary driver of plant autophagy functional network, we used these 997 genes to identify 92,967 AAG analogs among 77 plant species (Tables S7 and S8) (*p* < 1 × 10^−10^, amino acid identity >60%). For example, 1117 AAG analogs were identified in *Oryza sativa*, 1479 in *Solanum lycopersicum*, 1927 in *Camellia oleifera*, and 1387 in *Vitis vinifera*. Then, gene duplication events were detected using the MCScanX. All the AAG analogs were divided into five types: singletons (4659; 5.01%), dispersed genes (30,468; 32.77%), proximal duplicates (2733; 2.94%), tandem duplicates (12,629; 13.58%), and polyploidy‐related genes (42,478; 45.69%) (Figure 1G, Table S8). Notably, AAGs accounted for a significant increase in the proportion of all annotated genes in each genome during plant territorialization and AAGs were significantly overrepresented in the polyploid‐related group (*p* = 1.55E−15) and underrepresented in all other groups (Figure 1H, Table S9). In terrestrial plants, the predominant mechanism was polyploidization (*p* = 6.66E−16), with AAGs accounting for 1.19%–7.47% of all annotated genes in each representative genome (Figure 1H,I and Table S10). Overall, these findings suggest that polyploidization drives the formation of AAGs in plants.

Examination of three model terrestrial plants, *Arabidopsis thaliana*, *O. sativa*, and *S. lycopersicum*, indicated that AAGs were significantly overrepresented (*p* = 1.33E−15) in polyploid‐related groups, but underrepresented in all other groups (Figure S1B, Table S10). We used the M‐MAD tool to infer the autophagy co‐function of these three plant polyploid groups and compared these with human and yeast. Overlap analysis indicated that humans, yeasts, and plants shared 50 autophagy co‐functions, including cellular response to unfolded protein, lipid oxidation, and modification‐dependent protein catabolic process (Figure 2A, Table S11). Notably, humans and plants shared specific autophagy co‐functions that were not shared with yeasts (Figure 2A, Table S11). Plant‐specific autophagy co‐functions included chloroplast division, root hair elongation, and cell wall macromolecule catabolic process (Figure 2B, Table S11). We subjected these plant‐specific autophagy functions to correlation network analysis using the autophagy data set (GSE156677) and found that AAGs in the polyploid group associated plant‐specific chloroplast division with autophagy, while AAGs in other groups did not (Figure S1C, Table S12). Overall, it appears that AAGs in the polyploid associate plant‐specific functions such as chloroplast division with autophagy.

**Figure 2:**
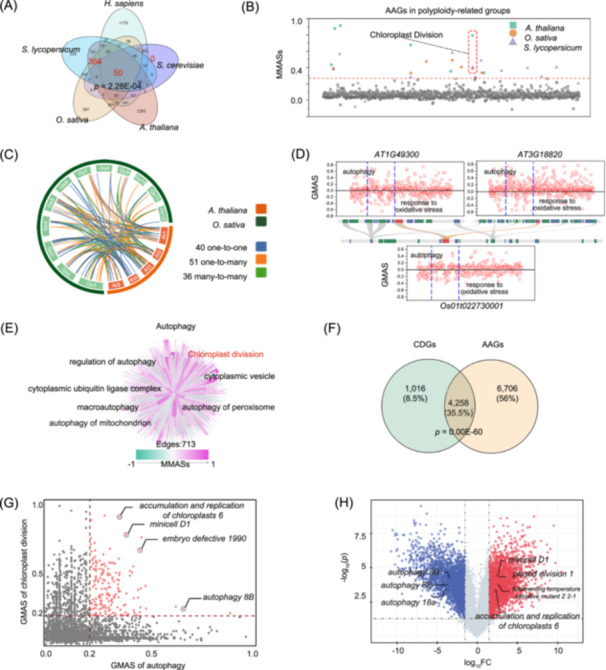
Functional differentiation of polyploidy autophagy‐associated genes (AAGs). (A) Venn diagram of autophagy‐related functions in *Arabidopsis thaliana*, *Oryza sativa*, *Solanum lycopersicum*, *Saccharomyces cerevisiae*, and *Homo sapiens*, based on Module‐Module Association Determination (M‐MAD). (B) Functions of AAGs in polyploidy‐related groups in *A. thaliana*, *O. sativa*, and *S. lycopersicum*, based on M‐MAD. Values are module‐module association scores (MMASs). (C) The phylogenetic relationship results of AAGs in *A. thaliana* and *O. Sativa* through TBtools [14]. Red: *A. thaliana*; Deep green: *O. Sativa*; Blue: one‐to‐one orthogroups; Orange: one‐to‐many orthogroups; Green: many‐to‐many orthogroups. (D) *Os01t022730001* of *O. sativa* forms an orthogroup with *AT1G49300* and *AT3G18820* of *A. thaliana*. This gene is expanded in *A. thaliana*, although its function in oxidative stress response is retained and redundant between the two genes. (E) Analysis of the autophagy co‐functions based on M‐MAD. The color depth indicates the MMASs of the co‐functional network of autophagy. (F) Venn diagram for shared genes analysis of chloroplast division genes (CDGs) and AAGs based on G‐MAD. (G) Shared genes between AAGs and CDGs. Each gene contains two gene‐module association scores (GMAS), with the *X*‐axis representing the |GMAS| of autophagy and the Y‐axis representing the |GMAS| of chloroplast division. Genes with |GMAS| > 0.2 for both the *X*‐axis and *Y*‐axis are defined as high‐confidence genes, represented by red points. (H) The significant differential expression genes in the data set (GSE184340) (*p* ≤ 0.05, fold change ≥ 1.5). Red indicates upregulated genes and blue indicates downregulated genes.

Additionally, taking *O. sativa* and *A. thaliana* as examples, we further compared the functional gains and losses of polyploid AAGs in monocotyledons and dicotyledons. The polyploid AAGs of *O. sativa* (434) and *A. thaliana* (936) formed 62 one‐to‐one single copy orthogroups, 69 one‐to‐many orthogroups, and 38 many‐to‐many orthogroups. Using the workflow in this study (Figure S1D), we obtained the co‐function of each gene, analyzed the functional overlap of orthogroups, and characterized the functional gains and losses of polyploidy AAGs. In the case of abiotic stress response, 81 AAGs in *A. thaliana* and 90 AAGs in *O. sativa* were found to participate in abiotic stress response process, forming 68 orthogroups (Figure 2C, Table S13). It should be noted that gene expansion often results in functional redundancy. For example, *Os01t022730001* of *O. sativa* formed an orthogroup with *AT1G49300* and *AT3G18820* of *A. thaliana*; indicating that this AAG is expanded in *A. thaliana*, and its stress response function is retained and redundant between the two genes (Figure 2D, Table S13) [15]. Besides, *mitogen‐activated protein kinase 3* (*MPK3*), *mucilage‐modified 1* (*MUM1*), and *ribosomal protein l23aa* (*RPL23AA*) have been confirmed to respond to oxidative stress in *A. thaliana*, but are lost in *O. sativa* [16] (Table S13).

The biological processes related to chloroplasts, such as the regulation of photosystem II, chloroplast division, and chloroplast starch grain, are unique functions of photosynthetic plants [17]. M‐MAD analysis has revealed that autophagy is associated with several chloroplastic functions, including chloroplast division (Figure 2E, Table S5). In our previous research, 5274 genes have been annotated as chloroplast division genes (CDGs) [18], including the known division genes *filamenting temperature‐sensitive mutant Z* (*FtsZ*), *accumulation and replication of chloroplasts 3* (*ARC3*), and *plastid division 1* (*PDV1*), as well as high‐confidence candidates such as *enhanced response to ABA 1* (*ERA1*) and *ethylene‐dependent gravitropism‐deficient and yellow‐green‐like 2* (*EGY2*), among others. Overlap analyses showed that CDGs and AAGs shared 4, 258 genes (Figure 2F, Table S14), including 179 high‐confidence genes (gene‐module association score, GMAS > 0.2) such as the established chloroplast division genes *accumulation and replication of chloroplasts 6* (*ARC6*), *minicell D1* (*MinD1*), *embryo defective 1990* (*EMB1990*), and autophagy gene *autophagy 8B* (*ATG8B*) [19] (Figure 2G). It was further found that the chloroplast division genes *ARC6*, *MinD1*, and *YLMG1‐1* were significantly expressed in *A. thaliana* (GSE184340) under autophagy state (Figure 2H, Table S15).

In summary, by constructing a co‐functional network of autophagy and reconstructing the evolutionary history of core AAGs, we can better understand how polyploidy promotes autophagy to participate in more plant‐specific functions. Polyploidization not only increases the number of AAGs in evolution but also affects the gain and loss of related functions. The interplay between chloroplast division and autophagy sheds light on the diversification of autophagic functions in plants, enhancing our comprehension of plant autophagy and offering fresh insights into the realm of plant autophagy research.

## AUTHOR CONTRIBUTIONS

**Moyang Liu**: Writing—original draft; formal analysis; data curation; methodology; software; conceptualization; funding acquisition; resources. **Ming Yang**: Writing—review and editing; formal analysis. **Heng Liang**: Funding acquisition; resources; writing—review and editing. **Bote Luo**: Visualization; validation. **Junjie Deng**: Visualization; investigation; formal analysis. **Lingyan Cao**: Visualization; validation; software. **Daojun Zheng**: Funding acquisition; writing—review and editing; supervision. **Cheng Chen**: Supervision; resources; writing—review and editing; writing—original draft; funding acquisition.

## CONFLICT OF INTEREST STATEMENT

The authors declare no conflicts of interest.

## ETHICS STATEMENT

No animals or humans were involved in this study.

## Supporting information


**Figure S1.** Workflow and gene function evolution based on multi‐omics data.

**Table S1.** Summary of data resources.
**Table S2.** Autophagy‐associated genes according to G‐MAD.
**Table S3.** Autophagy‐associated genes according to DAP‐seq data.
**Table S4.** Autophagy‐associated genes according to CF‐MS data.
**Table S5.** Autophagy co‐functions at different levels of regulation according to M–MAD.
**Table S6.** Correlation network of Autophagy pathway genes and AAGs.
**Table S7.** The information of plant genomes used in this study.
**Table S8.** AAG analogs identified in this study.
**Table S9.** The summary of five duplication types for each AAGs whole genome genes in 77 representative species.
**Table S10.** The significance analysis for each duplication type of AAGs in 77 representative species. The *p*‐values were obtained by Hypergeometric Distribution, and U and I represent un‐change and increase, respectively.
**Table S11.** Overlap analysis of autophagy‐related functions in five species according to M–MAD.
**Table S12.** Correlation network of Chloroplast division genes and AAGs.
**Table S13.** Functional differentiation of polyploidy AAGs in *A. thaliana* and *O. sativa*.
**Table S14.** Common genes in autophagy and chloroplast division.
**Table S15.** Differential expression analysis in GEO data (GSE184340).

## Data Availability

The public transcriptomic data of the focal species were downloaded from the National Center for Biotechnology Information NCBI (https://www.ncbi.nlm.nih.gov/). The authors also acquired DAP‐SEQ and CF‐MS datasets for *A. thaliana*, as well as 144 natural *A. thaliana* accessions (GEO: GSE43858 https://www.ncbi.nlm.nih.gov/geo/query/acc.cgi?acc=GSE43858), the *A. thaliana* 1001 Genomes Project (GEO: GSE80744 https://www.ncbi.nlm.nih.gov/gds/?term=GSE80744), and the OneKP Project transcriptome datasets (Table S1). Origin Pro 2021 software (OriginLab Corporation) was used to analyze experimental data and draw figures. Supporting Informaiton (methods, figures, tables, graphical abstract, slides, videos, Chinese translated version, and updated materials) may be found in the online DOI or iMeta Science http://www.imeta.science/.
