# Supplementary material for: Polyploidy drives autophagy to participate in plant‐specific functions
Source: Imeta. 2024 Dec 9;3(6):e252. doi: 10.1002/imt2.252 (PMC11683458; doi:10.1002/imt2.252)
Supplement: Supplementary file 1 — Figure S1. Workflow and gene function evolution based on multi‐omics data. [file IMT2-3-e252-s002.docx]

**Supporting information to**

**Polyploidy drives autophagy to participate in plant-specific functions**

**Running title:** Evolution of plant autophagy

Moyang Liu^1,3,4#^, Ming Yang^1,4^^#^, Heng Liang^2,3^^#^, Bote Luo^1,4^, Junjie Deng^1,4^, Lingyan Cao^5^, Daojun Zheng^2,3^^*^, Cheng Chen^1,3,4*^

^1^Shanghai Collaborative Innovation Center of Agri-Seeds/School of Agriculture and Biology, Shanghai Jiao Tong University, Shanghai 200240, China

^2^Institute of Tropical Horticulture Research, Hainan Academy of Agricultural Sciences, Haikou 571100, China

^3^Tropical Horticultural Plant Research Center, Hainan Research Institute, Shanghai Jiao Tong University, Sanya 572000, China

^4^Joint Center for Single Cell Biology, School of Agriculture and Biology, Shanghai Jiao Tong University, Shanghai 200240, China

^5^Joint International Research Laboratory of Metabolic and Developmental Sciences, State Key Laboratory of Hybrid Rice, School of Life Sciences and Biotechnology, Shanghai Jiao Tong University, Shanghai 200240, China

^#^These authors contributed equally: Moyang Liu, Ming Yang, Heng Liang

*Correspondence: [cgchen@sjtu.edu.cn](mailto:cgchen@sjtu.edu.cn) (Cheng Chen), [daojunzh@163.com](mailto:daojunzh@163.com) (Daojun Zheng)

**Supplemental Methods**

**Data sources and sequence retrieval**

The public transcriptomic data of the focal species were downloaded from the National Center for Biotechnology Information (NCBI). We also acquired DAP-seq and CF-MS datasets for *A. thaliana*, as well as 144 natural *A. thaliana* accessions (GEO: GSE43858), the *A. thaliana* 1001 Genomes Project (GEO: GSE80744), and the OneKP Project transcriptome datasets (Table S1).

**GeneBridge analysis**

In order to eliminate any potential confounding variables, we used the PEER tool to preprocess the data [1]. The data were then transferred to the GeneBridge toolkit for subsequent analysis. Ontology terms, biological pathways, and knowledge-based gene sets from diverse resources are all referred to as "modules" in the GeneBridge toolset, and only ontology terms were utilized in this work. In order to ascertain probable gene functions, the G-MAD tool in the GeneBridge toolkit utilizes expression data from large-scale cohorts. To ascertain the connections between relevant genes and biological modules, the Correlation Adjusted MEan RAnk gene set test (CAMERA), a competitive gene set testing technique used by G-MAD, makes allowances for inter-gene correlations [2]. A binding score of 1 or -1, depending on the direction of the enrichment, was given to gene-module interactions with enrichment *p*-values that persisted after several testing adjustments of the gene and modules; otherwise, they were assigned a binding score of 0. After performing a meta-analysis on the datasets, the average binding scores were weighted by the inter-genic correlation coefficient within modules ($\overline{p}$) and sample size to provide gene-module association scores.

Based on cross-species transcriptome compendia, the M-MAD tool in the GeneBridge toolkit was used to identify linkages between modules. The G-MAD results for each module against all genes were used to determine the relationships of genes against all modules. The gene-level data collected using the CAMERA technique were used to compute the enrichment against all modules using the enrichment scores of all genes for the target modules [2]. Based on Bonferroni thresholds, the condensed cross-module *p*-values were changed to 1, 0, or -1, and a meta-analysis was performed on all datasets to produce MMASs. Analysis of complete expression datasets was used to build the module association network. Previously published gene annotations were used to build the module similarity network. Therefore, the module association network permits the discovery of new biological linkages between modules that have not been previously documented in the literature.

**Identification of AAGs across 77 plant species genomes**

First, the genome sequences of 77 diverse plant species were downloaded (Table S7). Then, the 997 AAGs shared across the protein, transcription, and transcriptional regulation levels in *A. thaliana* were searched against protein sequences using the BlastP program, with an E-value threshold of 1 × 10^−10^ and amino acid identity > 60%. BlastP was performed on the identified sequences in the NCBI database to obtain annotation information. Finally, AAGs for all species were screened based on annotation information (Table S8).

**Identification of AAG duplication types**

The duplicate-gene-classifier program in MCScanX was used to ascertain different types of duplicated genes according to previous studies [3, 4], and the proportion of each special duplication event in the AAGs was calculated.

**Identification of multispecies orthologs**

The AAG orthologs in *A. thaliana* and *O. sativa* were inferred with OrthoFinder2, using default settings [5]. Multiple sequence alignments (MSAs) were inferred first, and then gene trees were inferred from these MSAs.

**Transcriptomic analysis**

The gene expression levels (BaseMean) of each sample were normalized using the DESeq2 package [6], and a threshold of padj (adjusted *p*-value) < 0.05 was used to identify DEGs for each comparison group.

**Construction and visualization of the correlation network**

The R package imsbInfer from GitHub was used to create the correlation network. Using Pearson's rank correlation measure, correlations were calculated. In the graph, positive correlations were indicated by red edges, while negative correlations were indicated by blue edges.

**Statistical analysis**

The experimental data were analyzed using Origin Pro 2021 software (OriginLab Corporation). Significant difference between groups were determined using least significant difference tests, and the thresholds for statistical significance were 0.05 and 0.01.

**References**

1. Stegle, Oliver, Leopold Parts, Matias Piipari, John Winn, Richard Durbin. 2012. “Using probabilistic estimation of expression residuals (PEER) to obtain increased power and interpretability of gene expression analyses.” *Nature protocols* 7: 500-507. <https://doi.org/10.1038/nprot.2011.457>

2. McWhite, Claire D., Ophelia Papoulas, Kevin Drew, Rachael M. Cox, Viviana June, Oliver Xiaoou Dong, Taejoon Kwon, et al. 2020. “A pan-plant protein complex map reveals deep conservation and novel assemblies.” *Cell* 181: 460-474.e414. <https://doi.org/10.1016/j.cell.2020.02.049>

3. Wang, Yupeng, Haibao Tang, Jeremy D Debarry, Xu Tan, Jingping Li, Xiyin Wang, Tae-ho Lee, et al. 2012. “MCScanX: a toolkit for detection and evolutionary analysis of gene synteny and collinearity.” *Nucleic Acids Research* 40: e49-e49. <https://doi.org/10.1093/nar/gkr1293>

4. Song, Xiaoming, Ying Li, Xilin Hou. 2013. “Genome-wide analysis of the AP2/ERF transcription factor superfamily in Chinese cabbage (*Brassica rapa* ssp. pekinensis).” *BMC Genomics* 14: 573. <https://doi.org/10.1186/1471-2164-14-573>

5. Emms, David, Steven Kelly. 2018. OrthoFinder2: fast and accurate phylogenomic orthology analysis from gene sequences. <https://doi.org/10.1101/466201>

6. Love, Michael I, Wolfgang Huber, Simon Anders. 2014. “Moderated estimation of fold change and dispersion for RNA-seq data with DESeq2.” *Genome Biol* 15: 550. <https://doi.org/10.1186/s13059-014-0550-8>


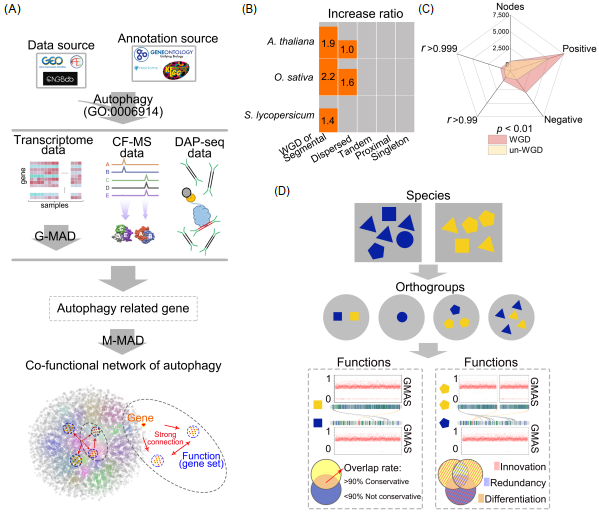


**Figure S1 Workflow and gene function evolution based on multi-omics data.** (A) Public databases were used to collect multi-omics data from diverse species. G-MAD, CF-MS, and DAP-seq were used to ascertain genes at the transcription, protein and transcriptional regulation levels. The genes identified at each level were used to determine the functions of the target via M-MAD. (B) The ratio of each gene classification in *Arabidopsis thaliana*, *Oryza sativa*, and *Solanum lycopersicum*: increased (orange, *p* < 0.01), or not significantly different (grey). (C) Correlation network of chloroplast division genes and WGD-AAGs (red) and unWGD-AAGs (yellow) (*p* < 0.01). (D) Schematic diagram of functional differentiation. The phylogenetic relationships of AAGs in different species were obtained by OrthoFinder2. The function of orthogroup-centralized genes was compared using G-MAD. A single-copy orthogroup with a gene functional overlap rate of more than 90% is considered conservative. The specific function of the genes in the multi-copy orthogroups is considered biological innovation. The shared function of repetitive genes within the same species is considered redundancy. Functions shared with other species or different within species are considered differentiation.
